# Retrograde tibial nailing of far distal tibia fractures: a biomechanical evaluation of double- versus triple-distal interlocking

**DOI:** 10.1007/s00068-021-01843-5

**Published:** 2021-12-03

**Authors:** Julia Greenfield, Philipp Appelmann, Felix Wunderlich, Dorothea Mehler, Pol Maria Rommens, Sebastian Kuhn

**Affiliations:** 1 Department of Orthopaedics and Traumatology, University Medical Centre of the Johannes Gutenberg University, Langenbeckstrasse 1, 55131 Mainz, Germany; 2 Department of Digital Medicine, Medical School OWL, Bielefeld University, Universitätsstr. 25, 33615 Bielefeld, Germany

**Keywords:** Distal Tibia Nail^®^, Double locking, Far distal tibia fracture, Biomechanical testing, Retrograde nailing

## Abstract

**Objectives:**

Retrograde tibial nailing using the Distal Tibia Nail (DTN) is a novel surgical option in the treatment of distal tibial fracture. Its unique retrograde insertion increases the range of surgical options in far distal fractures of the tibia beyond the use of plating. The aim of this study was to assess the feasibility of the DTN for far distal tibia fractures where only double rather than triple-distal locking is possible due to fracture localisation and morphology.

**Methods:**

Six Sawbones^®^ were instrumented with a DTN and an AO/OTA 43-A3 fracture simulated. Samples were tested in two configurations: first with distal triple locking, second with double locking by removing one distal screw. Samples were subjected to compressive (350 N, 600 N) and torsional (± 8 Nm) loads. Stiffness construct and interfragmentary movement were quantified and compared between double and triple-locking configurations.

**Results:**

The removal of one distal screw resulted in a 60–70% preservation of compressive stiffness, and 90% preservation of torsional stiffness for double locking compared to triple locking. Interfragmentary movement remained minimal for both compressive and torsional loading.

**Conclusions:**

The DTN with a distal double locking can, therefore, be considered for far distal tibia fractures where nailing would be preferred over plating.

## Introduction

**Fig. 1:**
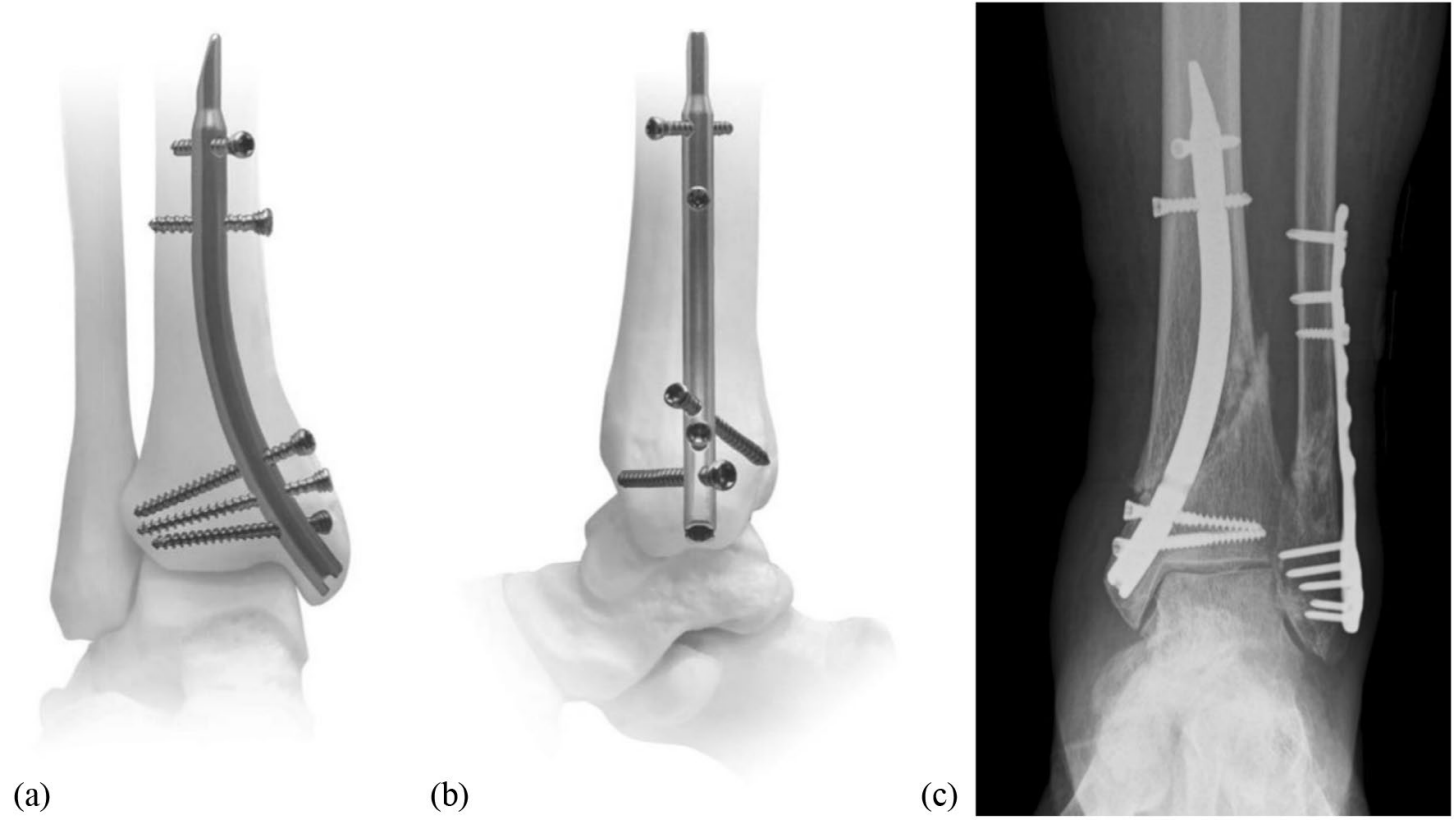
View of an implanted Distal Tibia Nail (DTN) in the antero-posterior (**a**) and medio-lateral (**b**) axes, and a patient radiography where only double-locking was possible distal to the fracture

Retrograde tibial nailing is a novel surgical option in the treatment of distal tibial fracture. Several attempts have been made in the past, dating back to Küntscher and Maatz in 1945 [1] as well as Hofmann in the late 1990s [2, 3]. However, until the introduction of the Distal Tibial Nail (DTN; Mizuho^®^, Japan) in 2017, no dedicated surgical implant was available [4]. Regarding this implant, three distal locking options are at 13, 19.5 and 26 mm from the nail end; the distal locking screws converge in the a.p. plane towards the tibial articular surface. In lateral view, the first and third locking screws diverge ± 25° compared to the second screw aligned at 0° (Fig. 1). Proximally, the DTN has two locking options at 88.5 and 95 mm. Possible indications for the DTN are, therefore, distal tibial shaft fractures (AO/OTA 42-A-C), distal extraarticular metaphyseal tibial fractures (AO/OTA 43 A1-3) and, in combination with an additional screw osteosynthesis, distal tibial fractures with simple intra-articular involvement (AO/OTA involvement (AO/OTA 43-C1). The DTN offers high biomechanical stability in distal tibia fractures due to its distal insertion site and multidirectional triple-distal interlocking. Additionally, the insertion of the end cap leads to an angle-stable distal locking screw to nail construct. It covers a wide range of fractures in the distal tibia [5], preserves local soft tissues and vascularisation, and offers superior biomechanical performance to plating [5] and equivalent biomechanical performance to other nailing options [6]. Following promising pre-clinical biomechanical testing results, the DTN was introduced into clinical practice in Japan in 2017 [4–6].

Far distal tibia fractures make up approximately 15% of all distal tibia fractures [7]. The cause of distal tibia fractures is related to high-energy trauma in young persons, while in the elderly it is more often due to a low-energy trauma, such as a fall [8]. Treatment of far distal tibia fractures presents a clinical problem [9]. Treatment options include open reduction and plate osteosynthesis, minimally invasive plate osteosynthesis (MIPO) or intramedullary (IM) nailing. The use of plating is subject to complications given the limited soft tissue coverage of the distal tibia [10, 11]. In the distal tibia, the use of plate osteosynthesis compromises vascularisation and soft tissues [12, 13]; in patients in which this area is already fragile, the use of plating must be critically assessed.

Antegrade IM nailing for far distal tibia fractures is associated with primary and secondary malalignment [14, 15], which jeopardises the reestablishment of the bone’s anatomical axis [16]. Furthermore, the far distal zone of the tibia (< 30–40 mm from the tibial plafond) cannot be reached by antegrade IM nailing [17–20]. Nonetheless, the use of an IM nail over a MIPO technique is beneficial if surgically feasible as the rate of infection, wound aggravation [20], and implant failure [21] are lower.

Even with this this novel implant, some far distal fracture configurations might only enable the purchase of the two most distal screws. The aim of this study was to conduct an exploratory analysis to assess to what extent double versus triple-distal locking of the DTN results in a reduction in stiffness construct and increase of interfragmentary movement.

## Materials and methods

### Sample preparation

Six composite tibiae (Sawbones^®^, Malmö, Sweden) samples were obtained for implantation. A detailed method of the DTN insertion can be consulted in Kuhn et al. [1]. Samples were proximally and distally embedded in PolyMethyl MethAcralate (PMMA) and subjected to low and high-level compression testing, described hereunder.

**Fig. 2:**
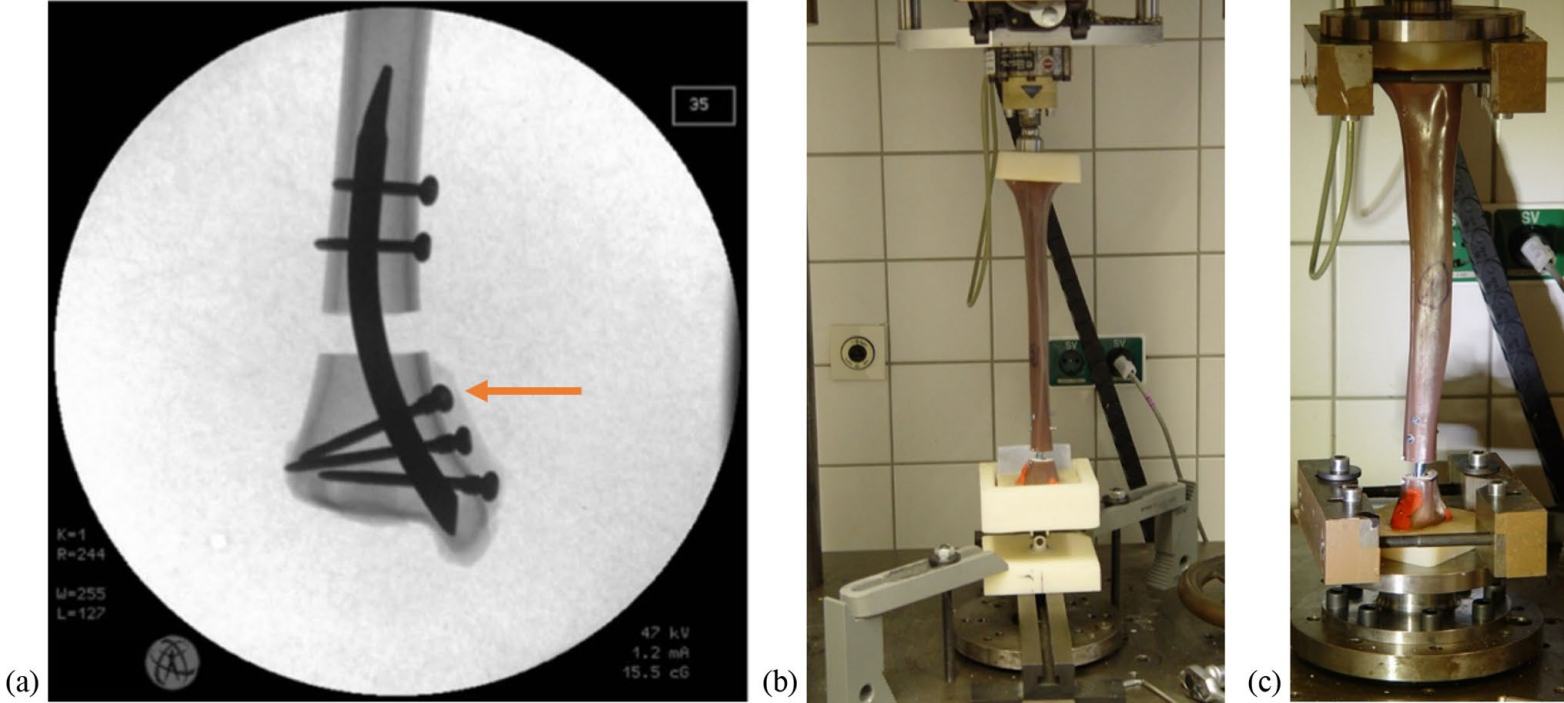
**a-c** AO/OTA 43-A3 fracture model with a 10 mm fracture gap located 40 mm above the tibial plafond (**a**), the arrow indicates the first distal locking screw that is to be removed in the case of a far distal fracture. Extra-axial compression test setup (**b**), and bidirectional torsion test setup (**c**)

An AO/OTA 43-A3 fracture presenting a 10 mm fracture gap located between 40 and 50 mm proximal to the tibial plafond (Fig. 2) was simulated in each of the samples. Fracture reduction was first carried out using two proximal screws and three distal locking screws (triple locking), the samples were then subjected to biomechanical testing. In the case of a far distal fracture, we considered that only the two most distal screws would be able to gain purchase in the bone (double locking). To test the stability of the DTN for far distal fractures, the most proximal of the distal screws was removed and the samples were then re-subjected to biomechanical testing.

### Biomechanical testing

The setup for biomechanical testing was based on Hansen et al. [22] and performed using a servopneumatic universal testing machine (SincoTec, Clausthal-Zellerfeld, Germany; acquisition frequency was set to 25 Hz) in the biomechanics laboratory of the University Medical Centre (Mainz, Germany). Samples were proximally and distally potted in PMMA, which improves their fit into the testing machine. Non-destructive extra-axial compression testing of 350 N and 600 N were carried out consecutively for each sample, followed by ± 8 Nm bidirectional torsional testing. An example of each test setup is shown in Fig. 2.

### Extra-axial compression

The extra-axial compressive loads were applied through a ball joint on the proximal PMMA, located 10 mm posteromedial from the centre axis of the tibia. At the distal end, the potted PMMA was placed in a universal joint (Fig. 2a). Following 18 N compressive pre-loading, extra-axial compressive loads of 350 N and 600 N were applied at a rate of 0.05 Hz over 24 cycles.

### Bidirectional torsion

For torsional testing, the proximal and distal ends on the sample, embedded in PMMA, were clamped into the testing machine (Fig. 2b). A compressive axial pre-load of 10 N and bidirectional torque of ± 8 Nm was applied to the distal end of the sample over 24 cycles. Torque–angle data were recorded by the moment and rotational transducers.

### Data analysis

Compressive and torsional stiffness construct were calculated from load–deformation curves. The first and last cycles were omitted from all stiffness calculations as these cycles presented anomalistic data. Stiffness calculations were based on 6 samples, each consisting of 23 cycles. Median stiffness construct and statistics were, therefore, based on 184 values. Torsional stiffness was calculated separately for positive and negative torque. Whole sample movement concerning linear and angular displacement data for compressive and torsional testing, respectively, were analysed during each test cycle.

Our exploratory analyses involved calculating double-locking stiffness as a percentage of triple-locking stiffness. No statistical analyses were carried out as the removal of one distal screw would certainly result in a reduction in stiffness; however, the aim of this study was not to prove as such, but to examine to what extent a reduction in stiffness is observed. For this reason, we found that the use of statistical analyses would be misleading.

## Results

**Fig. 3:**
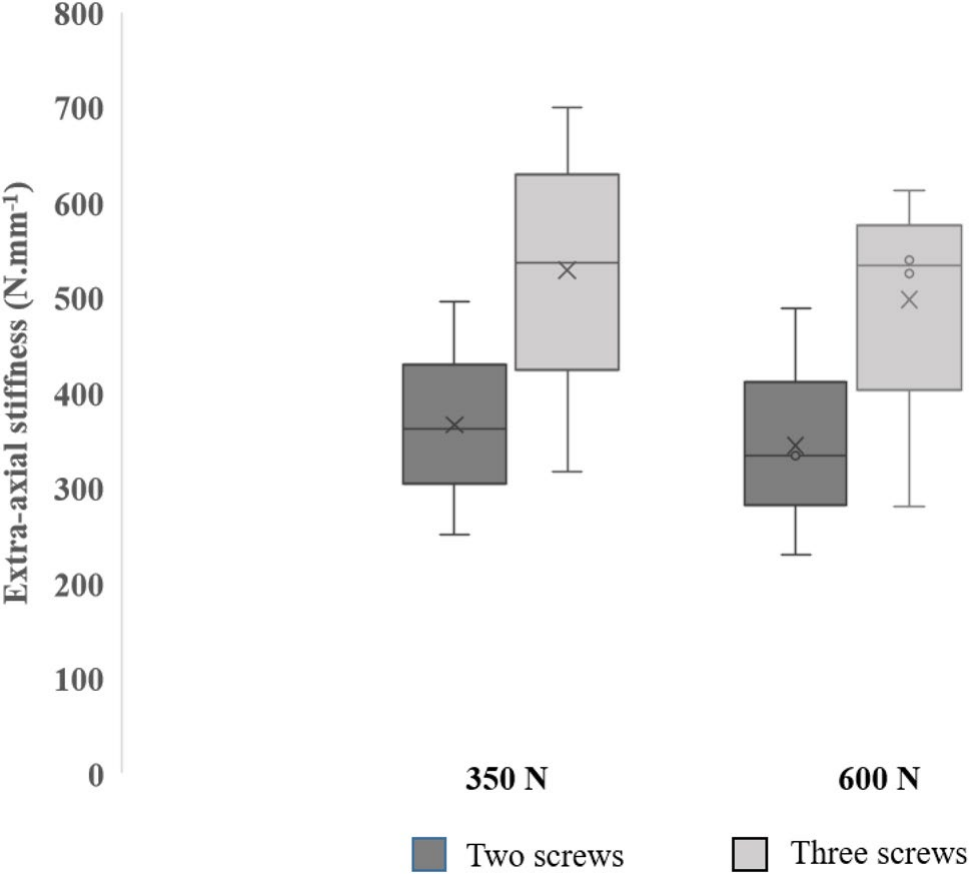
Box and whisker plot of stiffness construct at 350 N and 600 N extra-axial compressive loading for double-locking samples, compared to triple-locking

The following results are cited as the median (interquartile range) stiffness values for each sample group. Stiffness construct for compressive loading is greater at both 350 N and 600 N for the samples fixed with triple locking (Fig. 3). At 350 N, median stiffness constructs for double-locking samples are 359 (95) N.mm^−1^, for triple-locking samples it is 518 (129) N.mm^−1^, corresponding to a 69% preservation in stiffness construct in the double-locking configuration compared to triple locking. At 600 N, stiffness construct is very similar to 350 N in both sample groups, presenting a median of 338 (85) N.mm^−1^ for double locking, and 533 (118) N.mm^−1^ for triple locking, corresponding to a 63% maintenance in stiffness construct for the double-locking samples.

**Fig. 4:**
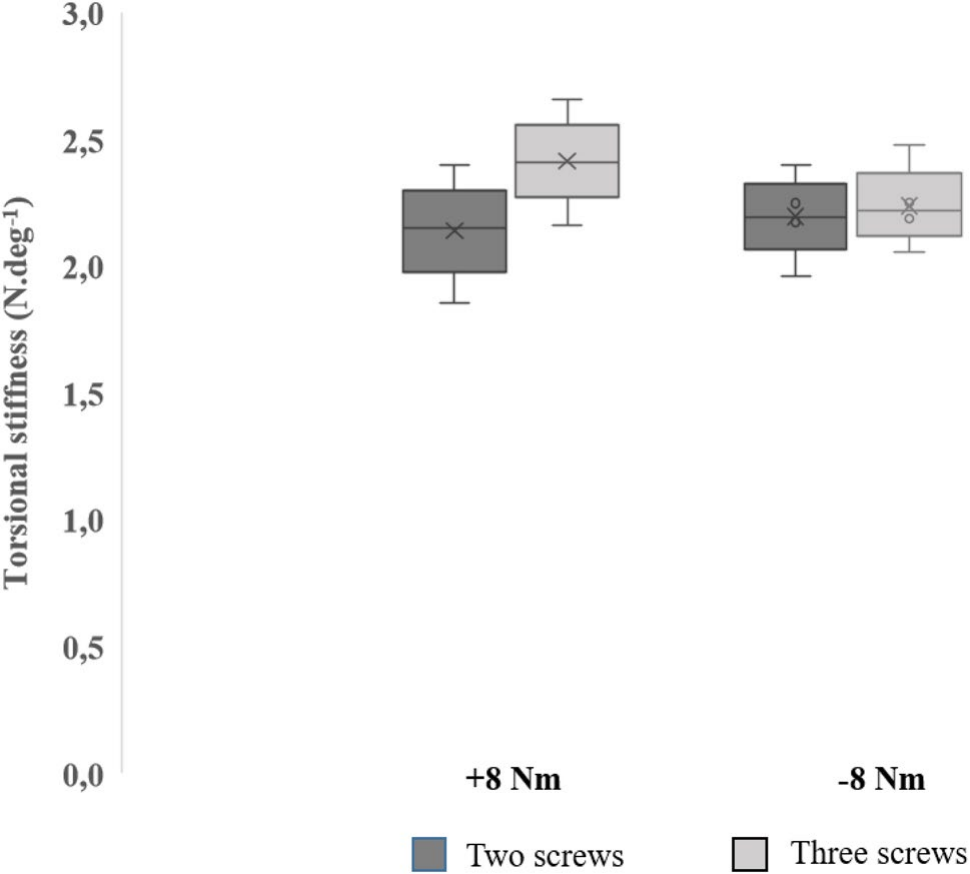
Box and whisker plot of stiffness construct at ± 8 Nm torsional loading for samples fixed with two distal screws, compared to three distal screws

As with extra-axial stiffness, torsional stiffness construct is greater in the triple-locking group for both positive and negative torsional loads (Fig. 4); however, the difference in torsional stiffness is minimal. For double-locking samples, median stiffness construct calculated during applied positive torsional loading was 2.14 (1.34) N.deg^−1^, and 2.40 (0.38) N.deg^−1^ for the triple-locking group, indicating a conservation of 89% stiffness in double locking. Negative torsional stiffness construct was calculated to be 2.22 (0.29) N.deg^−1^ for the double-locking samples, and 2.24 (0.27) N.deg^−1^ for the triple-locking group; this amounts to a 99% preservation of stiffness between sample groups.

**Fig. 5:**
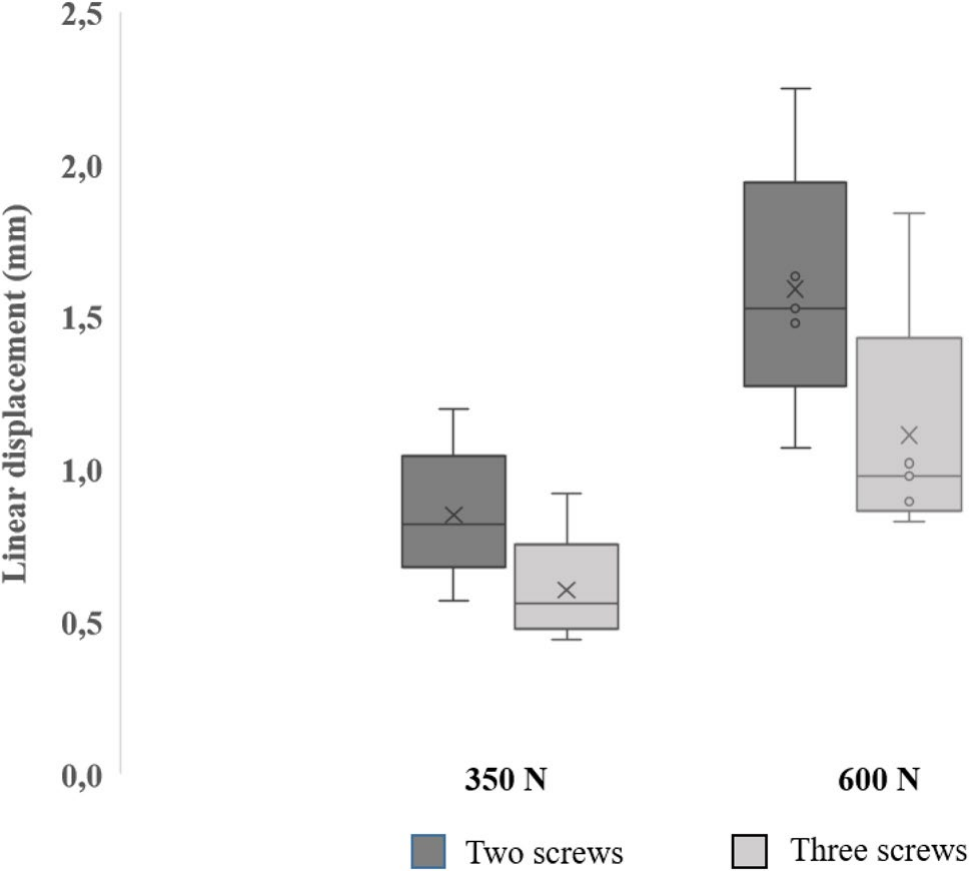
Box and whisker plot of interfragmentary movement at 350 N and 600 N extra-axial compressive loading for samples fixed with two distal screws, compared to three distal screws

Linear displacement measured by the testing machine displacement transducer shows a higher amount of movement for the samples fixed with two than with three distal screws (Fig. 5). At − 350 N loading, displacement averages at 0.84 (0.29) mm for the double-locking samples and 0.60 (0.01) mm for triple-locked samples; this demonstrates a 40% increase in linear displacement when one screw is removed. At − 600 N, 1.56 (0.56) mm is recorded for average linear displacement (increase of 41%) in double locking, and 1.11 (0.03) mm for triple locking.

**Fig. 6:**
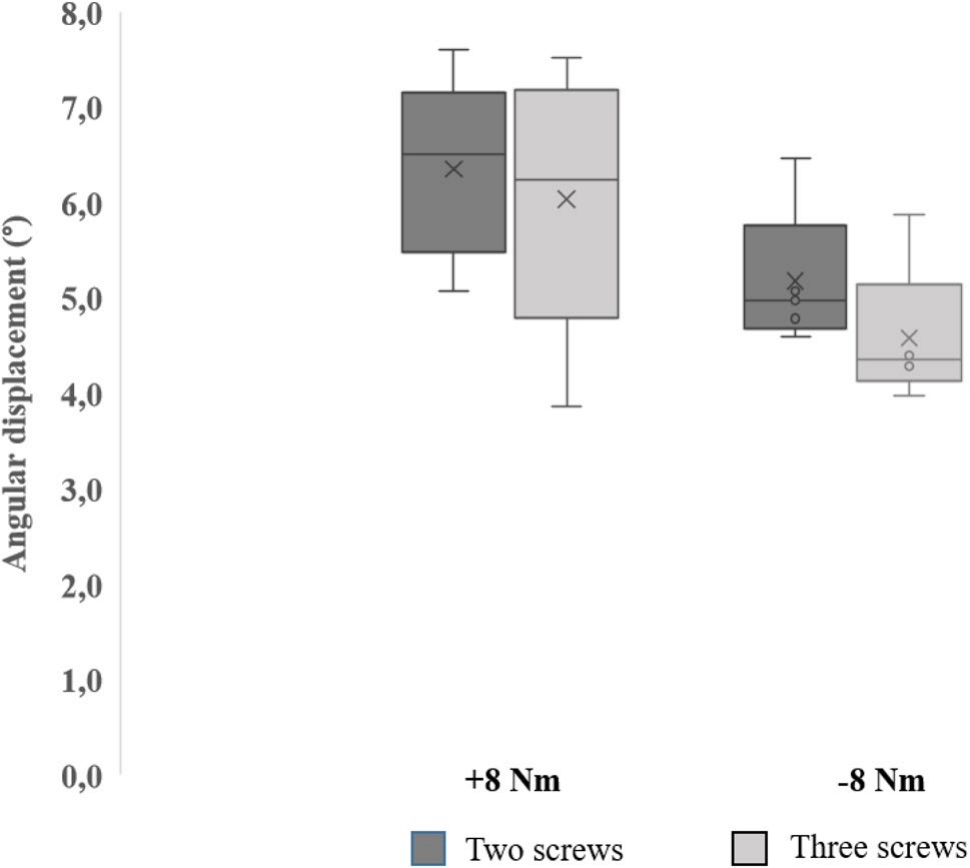
Box and whisker plot of interfragmentary rotation at ± 8 Nm torsional loading for samples fixed with two distal screws, compared to three distal screws

Angle data from the testing machine show an average of 6.33° (1.10) rotational movement (which meant an increase of 6%) for + 8 Nm torque application in double-locked samples (Fig. 6), and 5.96° (0.3) for samples with triple locking. For negative torque, double and triple-locking angular displacement values are 5.17° (0.96) and 4.64° (0.22), respectively; showing an increase of 11% in angular displacement for double versus triple locking.

## Discussion

Following previous biomechanical evaluation of the DTN in distal tibia fractures in previous studies [1–3], the aim of this study was to compare the use of the DTN in the triple-locking configuration with a double-locking configuration to assess the possibility of this implants use for far distal tibia fractures, which do not allow for triple locking. The results demonstrate a lower stiffness construct and increase in interfragmentary movement across all tests for fractures fixed with just two distal screws, which was expected with the removal of one interlocking screw. The removal of one load transfer interface (one screw) between the bone and the implant, applies a greater stress on the remaining bone–implant interface points for a given load, resulting in greater stress and strain applied to areas of the bone localised around the screws.

On closer inspection of the difference detected between the double and triple-locking groups, linear displacement of the samples was evaluated. This criterion gives an insight into the interfragmentary movement (IFM) allowed by each implant configuration. Despite the double-locking sample group presenting far higher linear displacement, the overall recorded displacement is low, falling below the 2 mm threshold often considered to be the level above which IFM is unacceptable [23–25]. Further, the linear displacement detected is based on the whole sample, including the PMMA embedding and test setup jig, and so one would expect the fracture gap movement to be even lower. The maximum difference in linear displacement between the two configurations was at − 600 N, where 0.55 mm greater movement was recorded for the double-locking configuration (resulting in a total of 1.56 mm linear displacement). It is important, however, to bear in mind that the A3 fracture defect studied here is a worst-case scenario in terms of stability and that for more stable fractures, less movement would be expected.

Results from the current study for the double-locking configuration of the DTN presents approximately 40% lower extra-axial stiffness construct values, and almost identical torsional stiffness values compared to a triple-locking configuration.

Previous studies on DTN prototypes have shown stiffness construct results within a similar range to those cited in this study for a triple-locking system. Kuhn et al. [1–3] cites axial stiffness values for 350 N compression testing ranging from 609 to 888 N.mm^−1^ and 627 to 797 N.mm^−1^ for 600 N compression testing. The higher extra-axial stiffness construct for standard triple-locking screw DTN fixation reported in these studies may be due to design modifications, for example related to nail length, which was shortened from a 120 mm prototype to the 116.5 mm final version between 2014 and the present study. Both the double- and triple-locking configurations in the current study present higher torsional stiffness construct (2.24 to 2.4 Nm.deg^−1^) compared to the aforementioned previous biomechanical evaluation studies, citing torsional stiffness constructs between 1.52 and 1.91 Nm.deg^−1^. This is also likely due to modifications made to the nail design after 2014; in particular the increase in divergence angle of the proximal screws from 30° to 45° with the aim of allowing less rotation when a torque is applied. Likening the results from the current study to those from previous studies in our research group (Biomechanics Laboratory of the University Medical Centre Mainz, Germany, [4 – 6]) demonstrates test setup reliability, facilitating the direct comparison of results.

Furthermore, despite the test setup being identical, minor differences in boundary conditions can play an important role [26]. Although not quantified, sample and setup related factors such as PMMA embedding thickness, load application point, and test setup stiffness may result in differing results between samples.

Differences in torsional stiffness constructs were high between double- and triple-locking sample groups; but amount to a disparity of 0.63° between the two groups at − 8 Nm torsion. Median detected angular displacement was around 6° for all samples and configurations. To the author’s knowledge, there is no current evidence stating an acceptable level of rotation for fractures fixed with an implant, but it is widely considered that excessive torsional movements are harmful to the bone remodelling process [27–30]. As the triple-locking DTN is a biomechanically validated implant with respect to its permitted shear movement, we assume that the greater torsional stiffness in this study with both double and triple-locking screw configurations would remain largely acceptable, if not advantageous.

Data reported from previous studies concerning relevant implant options for A3 fracture fixation are summarised in Table 1. Standard deviations are cited in Table 1 where possible; when not cited, this information was not made available in the literature. The data from the present study are presented as mean stiffness construct ± standard deviation, rather than the median followed by the range, to facilitate the comparison with means from other studies.

**Table 1:** A summary of testing methods and results from previous work relating to the use of implants from A3 distal tibia factures, based on stiffness construct and interfragmentary movement

Author	Bone sample	Implant	Compression			Torsion		
			Compressive loads (N) [loading frequency]	Mean stiffness construct (N.mm^−1^) ± SD	IFM (mm)	Torsional loads (Nm) [loading frequency]	Stiffness construct (Nm.deg^−1^)	IFM (°)
Current study 2 screws	4th generation Sawbones^®^	DTN	18–350 [0.05 Hz]	369 ± 6	0.84 ± 0.29	± 8 [0.05 Hz]	+ 8 Nm: 2.15 ± 0.08 − 8 Nm: 2.21 ± 0.06	+ 8 Nm: 6.33 ± 1.11 − 8 Nm: 5.17 ± 0.96
			18–600 [0.05 Hz]	348 ± 2	1.57 ± 0.56			
Current study 3 screws	4th generation Sawbones^®^	DTN	18–350 [0.05 Hz]	533 ± 19	0.6 ± 0.01	± 8 [0.05 Hz]	+ 8 Nm: 2.41 ± 0.05 − 8 Nm: 2.26 ± 0.06	+ 8 Nm: 5.96 ± 0.3 − 8 Nm: 4.64 ± 0.22
			18–600 [0.05 Hz]	495 ± 7	1.11 ± 0.03			
Kuhn et al. 2014a [4]	4th generation Sawbones^®^	DTN	18–350 [0.05 Hz]	888 ± 192	0.39	± 8 [0.05 Hz]	+ 8 Nm: 1.83 ± 0.26 − 8 Nm: 1.83 ± 0.33	± 8 Nm: 4.37
		MIPO		213 ± 70	1.64		+ 8 Nm: 0.35 ± 0.08 − 8 Nm: 0.39 ± 0.03	+ 8 Nm: 22.86 − 8 Nm: 20.51
Kuhn et al. 2014b [5]	4th generation Sawbones^®^	DTN	18–350 [0.05 Hz]	609 ± 149	0.57	± 8 [0.05 Hz]	+ 8 Nm: 1.52 ± 0.1 − 8 Nm: 1.6 ± 0.11	+ 8 Nm: 5.26 − 8 Nm: 5
			18–600 [0.05 Hz]	627 ± 113	0.96			
		ETN	18–350 [0.05 Hz]	888 ± 249	0.39		+ 8 Nm: 0.68 ± 0.06 − 8 Nm: 0.66 ± 0.04	+ 8 Nm: 11.76 − 8 Nm: 12.12
			18–600 [0.05 Hz]	773 ± 229	0.45			
Kuhn et al. 2014c [6]	4th generation Sawbones^®^	DTN	18–350 [0.05 Hz]	870 ± 194	0.14 ± 0.03	± 8 [0.05 Hz]	± 8 Nm: 1.91 ± 0.21	± 8 Nm: 4.19
			18–600 [0.05 Hz]	929 ± 104	0.26 ± 0.05			
Snow et al. 2008 [31]	Osteoporotic synbone^®^	MIPO	450 [NS]	1750	3	3.5	0.17	22.34
Högel et al. 2012 [32]	4th generation Sawbones^®^	MIPO	0–350 [10 mm/min]	466 ± 50	1.03 ± 0.16	+ 5 to − 10 [18 deg/min]	0.59	15

*MIPO* minimally invasive plate osteosynthesis; *DTN* Distal Tibia Nail; *ETN* Expert Tibia Nail; *SD* standard deviation; *IFM* Interfragmentary movement; *NS* not stated

The other, more frequently used nailing option for distal tibia fractures is the Expert Tibia Nail (ETN; Synthes^®^, Switzerland). This implant leaves a gap of 10–15 mm between the nail end and the tibial plafond [3] and distal locking options are spaced at 5 mm, 13 mm, 37 mm (medio-lateral directed screws), and 22 mm (directed antero-posteriorly) from the distal tip of the ETN; a minimum of one medio-lateral and one antero-posterior screw is required to ensure stable interlocking. Nonetheless, given the necessity for a minimum of two locking screws, and considering the end of the medullary canal, the position of the ETN within the distal fracture fragment and the gap between the, respectively, employed screw holes, the zone including 25–30 mm of bone proximal from the tibial plafond cannot be treated.

For plating options, Kuhn et al. [2] measured the medial distal tibia plate (MDTP) against the DTN prototype in a test setup using a proximal spherical joint and a cardan joint at the distal end. The MDTP averaged at 213 N.mm^−1^ and 0.39 Nm.deg^−1^ for axial and torsional stiffness, respectively. One other study [32] having measured the biomechanical performance of locking plates for A3 fractures report average axial and torsional stiffness constructs of 466 N.mm^−1^ and 0.59 Nm.deg^−1^, respectively, in a similar test setup. The double-locking results from the current study demonstrate a similar magnitude of axial stiffness construct and up to four times higher torsional stiffness construct. Again, these results go in favour of the DTN’s use as a nailing option for far distal tibia fractures.

From a clinical perspective, the use of IM nailing is beneficial compared to plating as it has less impact on soft tissues around the fracture site and, therefore, this and vascularisation are not compromised decreasing the risk of co-morbidities [13, 33]. The geometric characteristics of the MDTP and its positioning make it more susceptible to bending forces during compression of the tibia in weight-bearing activities [34]. As a consequence, screw loosening is a common post-operative problem [35, 36] with the plate leading to implant instability and mechanical hardware protrusion through the skin. Furthermore, this procedure is generally avoided where possible in patients presenting an already compromised vascularisation around the distal tibia as the application of the plate only worsens this situation by applying pressure to the peri-bone blood vessels.

Removing a distal locking screw may increase the risk of screw loosening and compromise implant stability in the long term. To test this phenomenon against other implant options for far distal tibia fractures, it would be necessary to carry out cyclic testing over, for example, 10,000 cycles at a frequency of 1–2 Hz to simulate human walking [37].

Overall, the biomechanical data suggests a clinical use DTN in far distal tibial fractures even when only double interlocking is possible. Also, the principals of functional after treatment would remain unchanged to standard treatment. It allows for early active motion. Depending on fracture healing, partial weight bearing can be started after 6 and full weight bearing is expected 12 weeks after surgery [38].

## Conclusion

Double as opposed to triple-locking results in a preservation of 60–70% axial stiffness and 89–99% torsional stiffness. Based on these values, the functional after treatment of a patient would remain unchanged. Hence, we can conclude that, in the case of a far distal tibia fracture, the use of a double- rather than triple-locking system for the DTN can be considered.
